# Journal editors and academic medicine

**DOI:** 10.3325/cmj.2022.63.407

**Published:** 2022-10

**Authors:** Ana Marušić, Matko Marušić

**Affiliations:** 1 Department of Research in Biomedicine and Health, Center for Evidence-based Medicine, University of Split School of Medicine, Split, Croatia *ana.marusic@mefst.hr*; 2 Emeritus Professor, Founding and emeritus editor, ST-OPEN, University of Split, Split, Croatia

Twenty years ago, the *Croatian Medical Journal* (*CMJ*) published a series of articles about academic medicine and the ways to revitalize it in the context of contemporary challenges in medicine (1,2). The articles were from all around the world (Albania, Australia, Austria, Belgium, Bosnia and Herzegovina, Canada, Croatia, Denmark, Estonia, Finland, Germany, Hungary, Israel, Republic of Macedonia, Malawi, Poland, Russia, Slovenia, Switzerland, United Kingdom, and USA) and discussed the future of academic medicine as the intersection of research, professional practice, and education (1,2). Figure 1 shows the cover page of the *CMJ* from 2004, when the FORUM series on academic medicine was started.

**Figure 1:**
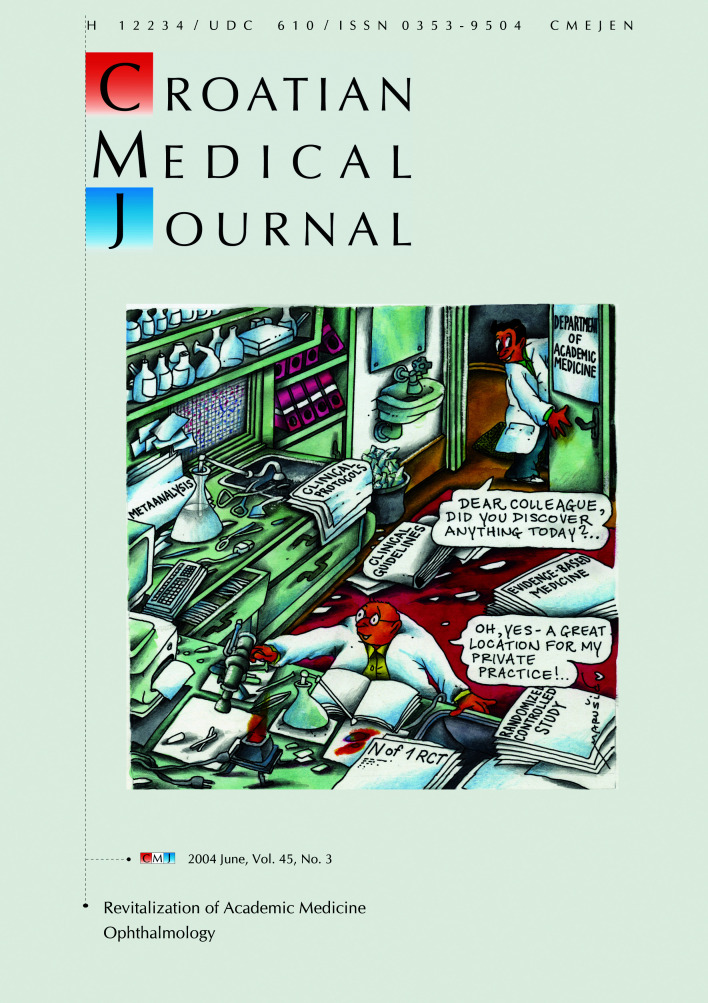
The cover page of the June 2004 issue of the *Croatian Medical Journal*.

The discussion about the future of academic medicine was initiated in 2003, when the *BMJ* invited a number of stakeholders to participate in a project called the International Campaign to Revitalise Academic Medicine (ICRAM). The *CMJ* was invited to join this initiative together with other journals, including *BMJ* specialist journals, *The Lancet*, C*anadian Medical Association Journal, Dutch Journal of Medicine*, and *Medical Journal of Australia* (1). The expert group discussed the future of medicine in the 21st century, the impact of academic medicine on health and health care, the position of academic medicine in the global professional and scientific community, and the ways to increase recruitment to and job satisfaction in academic medicine.

The group (with AM as the representative from the *CMJ*) used the methodology of scenario building to envisage how academic medicine would look like in the 21st century (3-5). We considered the contemporary problems and identified drivers for the change in future. This helped us to create five possible scenarios of plausible but different futures. We hoped the academic community would use these scenarios to reflect on the present and discuss the best way to develop academic medicine (5).

As the first quarter of the 21st century approaches its end, it is perhaps a good time to consider which of the futures seem closer to reality. Here are possible futures of academic medicine for 2024 as we saw them in 2003.

## Scenario 1: Academic Inc.

This scenario sees academic medicine primarily in the private sector, where teaching, practice, and research in medicine are privatized, governed by corporations. It is focused on market needs and efficiency. The Matthew effect of accumulated advantage (6) is strong: the rich systems get richer and the poor stay poor.

## Scenario 2: Reformation

There is no academic medicine. Everyone in the health care system learns, teaches, researches, and improves the system. It is led by societies of professionals and patients. Translational research is preferred, and health research is integrated with health care quality improvement.

## Scenario 3: In the public eye

This is consumer academic medicine – everything is gauged to please patients, the public, and the media. Medicine lives in the media and is governed from the bottom up, by patients. Expert patients teach.

## Scenario 4: Global academic partnership

This is academic medicine of global cooperative networks devoted to bridging the gap between the developed and developing world. Medical education and research is centered on global health, and teaching is done in partnership between medical schools from diverse settings. It is governed by institutional networks, with involvement of policy makers and the public.

## Scenario 5: Fully engaged

This is academic medicine fully engaged with and governed by all stakeholders. Medical training is community based, and students have an important say in setting educational goals. Research is inclusive, engaging individuals with different skills, including stakeholders such as patients, policymakers, students, etc.

## Which scenario came true?

None of the five scenarios prevailed. This is good, as any of the full scenarios is in contrast with the very character of medicine as a wide field, ranging from natural sciences (eg, chemistry – development of new more efficient and less dangerous drugs; physics – robotics and artificial intelligence) to medicine’s role of preserving human health and treatment of diseases (eg, smart drugs, personalized medicine) but also to its social, economic, political/policy, and even philosophical (eg, end-of-life decisions, gender health issues) character and impact. At the end of 2022, we can see that elements of all five scenarios have contributed to the present.

*Academic Inc.* is slow to come as most of the universities with medical schools are public or not-for-profit institutions, even the richest ones, such as Harvard University. However, profit is becoming an ever greater priority for all universities, and medical schools are often set within a private health care institution. In Croatia, both education and health care systems have been public. With the recent establishment of a medical school at a private university (7), it remains to be seen how the private sector will contribute to academic medicine in Croatia.

The *Reformation* scenario may have progressed the most: all stakeholders in academic medicine have become more involved in medical research, teaching, and practice, which certainly improves the system. Translational research has embraced the concept of personalized medicine. This contributed to the integration of health research with health care and led to higher quality of care and better health care outcomes.

*In the public eye* scenario, in which everything would be gauged to please the patients, the public, and the media, luckily, did not occur. Medicine is too important and knowledge-driven to “live” in the media. The media are, however, important in the transfer of knowledge from academic medicine to the public so that the new knowledge produced by academic medicine can improve health care (8).

*Global academic partnership* has grown significantly through the establishment of collaboration networks across the globe, supported by different funders (9). The COVID-19 pandemic demonstrated the importance of global collaborations and exchange of knowledge to inform health policies in rapidly changing epidemiological circumstances (10).

*Fully engaged* academic medicine would mean that it is fully engaged with and governed by all stakeholders. This is not the case with most academic medicine settings, but the concepts and elements are around us (11). Medical training is often community based, and students have an important say in setting educational goals. Research includes experts with different skills who come from different stakeholder groups – patients, policymakers, and students.

## What will happen in the next quarter of the century?

What is your view of academic medicine in future? We propose that the *Croatian Medical Journal* opens a discussion about how academic medicine will (or should) look in 2050 and collects the opinions of the relevant experts.
